# Suit for Malpractice

**Published:** 1851-07

**Authors:** 


*Suit for Malpractice. Prof. Spencer on Mercury in Dysentery.*—A suit for malpractice was recently brought against Prof. Thomas Spencer, formerly connected with Geneva Medical College, and now a resident of Milwaukie, Wisconsin, for alleged unskillful treatment of a case of dysentery, negligence, and the administration of calomel in large doses, in consequence of all which, as was claimed, the loss of teeth and destruction of a portion of the upper jaw ensued. The patient was a girl between four and five years of age.

The ground taken by the defense, and established by medical and other testimony, was,—"1. That no calomel was administered in any doses whatever, large or small; 2. That if calomel had been administered, it would have been accordant with high medical authority, and that it was almost the uniform practice of the medical profession to give calomel; 3. That all needful attention was given to the case by Dr. Spencer, because it was a case of simple necrosis; because nature, and not the surgeon, was the most important agent in the cure of such cases."

The verdict was for the defendant. After the conclusion of the trial, a meeting of the medical profession of Milwaukie took place, and congratulations were tendered to Prof. S., who responded by a brief address, in which he takes occasion to give his views respecting the employment of mercury in the treatment of acute dysentery. He says:

"I must confess to the indulgence of a just pride in the science of a profession I have for so long a period assumed to teach. But it was not only in this region of the world, but under the tropics, that I have had opportunity of verifying, by careful observation and experience, the principles of treatment I have for years promulgated in acute dysentery. This disease has been the scourge of the armies of our mother country, in ages gone by, and has alike been the scourge of our own. From the 8th of June to the 1st of July, upon the first arrival at Matamoras, Mexico, 507 cases of disease occurred in the 10th Regt., of which between three and four hundred were dysentery and kindred diseases, and the sole care of all without assistant surgeon or educated apothecary, was cast upon me as the medical prescriber. Notwithstanding this large number treated, but 5, either in June or the two succeeding months, fell victims to this scourge of armies, while, in the Virginia Regiment during the same period, as I was informed by Dr. Cobb, its able surgeon, about 90 fell victims to this class of diseases. Not only this Virginia Regiment, but others as points of comparison, where calomel was employed by army surgeons, convinced me, that the principles and practice I so long put in requisition, in this region, were alike applicable under the tropics.

"I do not believe that partiality, a natural partiality to my own views, has brought my mind to these conclusions, because if I love self, I love medical truth more. I have been most fortunate, on this occasion, to have been surrounded by those who, with great credit to themselves, had listened to my first, last, and many intermediate courses of instruction, and who could bear testimony to my uniform denunciation of calomel as a remedy in the dysentery of children. In this, I may have been a little ultra; but no matter for that; my views were put forth, and ample experience — my own and that of others — has tested their accuracy. Far be it from me to complain, or to have complained, of this prosecution. It does the profession good to bring them up as I have been. It sets them studying their profession, and this trial has given the evidence that not only those who have gone forth among a confiding community, from under my own teaching, but all who have testified on this occasion, have given evidence that they have kept pace with the rapid progress of our science."

At the meeting referred to, a committee was appointed to prepare a report of the trial for publication in some medical Journal.

The subject of suits for malpractice, is one in which the medical profession has not a small interest. In several points of view it presents important considerations. We had designed to have made the above case a text for an article on the subject, and in fact had advanced somewhat in its composition, when, finding that we had entered on a field too extended for the time and space at our command at the present time, we have concluded to place this on the list of editorial topics deferred for a "more convenient season."

